# Supplementary material for: WordCommentsAnalyzer: A windows software tool for qualitative research
Source: F1000Res. 2018 Sep 4;7:536. Originally published 2018 May 3. [Version 2] doi: 10.12688/f1000research.14819.2 (PMC6137411; doi:10.12688/f1000research.14819.2)
Supplement: Supplementary file 3 [file f1000research-7-17614-s0002.tgz › b2a79496-4ada-47c4-9682-a99ce867836d.zip!/Eight Iranian Health Journals/Journal of Education and Community Health_5.docx]

© Journal of Education and Community Health. This work is licensed under CC BY-NC 4.0 (https://creativecommons.org/licenses/by-nc/4.0/).

Volume & Issue: دوره 3 - شماره 4

Title: مطالعه خانواده من، برنامه پیشگیری‌کننده از شروع زودرس مصرف مواد: کاربرد رویکرد نقشه‌نگاری مداخله

http://jech.umsha.ac.ir/article-1-249-fa.html

سابقه و هدف: با توجه به پژوهش‌های صورت گرفته، مصرف مواد یکی از مسائل سلامت جامعه ایرانی است و علاوه بر افزایش شیوع، سن شروع مصرف نیز کاهش یافته و به اوایل نوجوانی و حتی کمتر رسیده است. هدف از این پژوهش، توسعه برنامه خانواده‌محور پیشگیری‌کننده از شروع زودرس مصرف مواد در کودکان (مطالعه خانواده من) با بهره‌گیری از رویکرد برنامه‌ریزی نقشه‌نگاری مداخله بود. مواد و روش‌‌ها: پژوهش حاضر پروتکل مطالعه‌ای است که در آن رویکرد نقشه‌نگاری مداخله به ‌عنوان چارچوب برنامه‌ریزی برای توسعه برنامه مطالعه خانواده من استفاده شد. در این مطالعه شش گام نقشه‌نگاری مداخله تکمیل گردید. اساس نیازسنجی مصاحبه با متخصصان و بررسی ادبیات مرتبط بود. در گام دوم از تقاطع اهداف عملکردی و تعیین‌کننده‌های مرتبط در ماتریکس‌ها، اهداف تغییر نگارش شدند. در ادامه، پس از طراحی برنامه و برنامه‌ریزی پیاده‌سازی مداخله، طرح ارزشیابی برنامه تکمیل گردید. یافته‌ها: استفاده از رویکرد نقشه‌نگاری مداخله ما را در توسعه یک برنامه نظامند، نظریه و شواهدمحور، همچنین مشخص ساختن پیامدها، اهداف عملکردی و تغییر، تعیین‌کننده‌ها، روش‌های تغییر نظری و کاربردهای عملی، برنامه مداخله، انتشار و ارزشیابی گام به گام راهنمایی و کمک کرد. نتیجه‌گیری: نقشه‌نگاری مداخله یک رویکرد نظامند، نظریه و شواهد محور به ‌منظور توسعه برنامه ارتقاء سلامت با کیفیت ادامه‌دار را فراهم می‌کند.

Title: بررسی تأثیر آموزش مبتنی بر مدل اعتقاد بهداشتی بر رفتارهای پیشگیری‌کننده از بیماری تب مالت در دامداران سنتی مناطق روستایی استان همدان

http://jech.umsha.ac.ir/article-1-176-fa.html

سابقه و هدف: آموزش رفتارهای پیشگیری کننده از بیماری تب مالت برای کاهش موارد ابتلا در گروههای در معرض خطر بیماری یک ضرورت است. بنابراین مطالعه حاضر با هدف تعیین تأثیر آموزش مبتنی بر مدل اعتقاد بهداشتی بر رفتارهای پیشگیری کننده از بیماری تب مالت در دامداران سنتی مناطق روستایی استان همدان انجام گردید. مواد و روش ­ها: این پژوهش یک مطالعه مداخله ای از نوع پیش آزمون-پس آزمون همراه با گروه کنترل بود که در سال 1394 بر روی دامداران سنتی روستاهای استان همدان که به عنوان کانون بیماری تب مالت  شناسایی شده بودند، انجام گردید. در این مطالعه 70 دامدار به طور تصادفی انتخاب و در دو گروه آزمون و کنترل تقسیم شدند. داده­ها با استفاده از پرسشنامه­ای مشتمل بر اطلاعات جمعیت شناختی، آگاهی، چک لیست رفتار و سازه­های مدل اعتقاد بهداشتی گردآوری شد. مداخله آموزشی شامل 4 جلسه آموزشی همراه با نمایش فیلم و استفاده از لوح تصویری و ارسال پیامک بود که در بین شرکت کنندگان گروه آزمون برگزار گردید. داده­ها با استفاده از آزمون­های کای اسکوئر، دقیق فیشر، تی­مستقل و تی زوجی مورد تجزیه و تحلیل قرار گرفت. یافته ­ها: نتایج نشان داد که بعد از مداخله آموزشی میانگین نمرات آگاهی، حساسیت درک شده، شدت درک شده، منافع درک شده، خودکارآمدی درک شده، راهنما برای عمل و رفتارهای پیشگیری کننده از تب مالت در گروه آزمون نسبت به گروه کنترل افزایش معنی­داری یافته بود (P<0.001). نتیجه­ گیری:نتایج مطالعه حاضر نشان داد که مداخله آموزشی بر اساس مدل اعتقاد بهداشتی می­تواند در بهبود رفتارهای پیشگیری کنده از تب مالت در دامداران سنتی مؤثر باشد.

Title: اثربخشی آموزش مفاهیم انیگرام بر ملاک‌های همسرگزینی و نگرش نسبت به ازدواج دختران

http://jech.umsha.ac.ir/article-1-244-fa.html

سابقه و هدف: بنیان و اساس اجتماع بر تشکیل خانواده بنا نهاده شده است. اما ازدواج یک مسئله‌ پیچیده و چالش‌برانگیز بوده که با توجه به عوامل مختلف از جمله نگرش به ازدواج می تواند موفق یا ناموفق باشد. این پژوهش با هدف تعیین اثربخشی آموزش مفاهیم نه گانه شخصیتی انیگرام بر ملاک‌های همسرگزینی و نگرش نسبت به ازدواج دختران مجرد انجام شد. مواد و روش ها: پژوهش حاضر یک مطالعه نیمه تجربی از نوع پیش آزمون-پس آزمون همراه با گروه کنترل بود. جامعه آماری این پژوهش شامل کلیه‌ دانش‌آموزان دختر مقطع سوم دبیرستان منطقه آموزشی 14 تهران در سال تحصیلی 95-1394 بود. در این مطالعه به روش نمونه گیری خوشه چندمرحله ای 2 مدرسه از این منطقه انتخاب و در نهایت 30 نفر از دانش‌آموزان این مدارس انتخاب و به صورت تصادفی در دو گروه آزمون و کنترل جایگزین شدند. داده ها از طریق پرسشنامه های اولویت بخشی ملاک‌های همسرگزینی (PCSSI) و نگرش به ازدواج (MAS) جمع آوری گردید. گروه آزمون تحت آموزش 10 جلسه 90 دقیقه ای یکبار در هفته قرار گرفت. داده ها با استفاده از نسخه 22 نرم‌افزار SPSS و به روش کوواریانس تحلیل شدند. یافته ها: نتایج نشان داد که بین نمره نگرش کلی و نگرش بدبینانه به ازدواج و ملاک‌های کلی، فرآیندی و محتوایی همسرگزینی دو گروه آزمون و کنترل در پس آزمون تفاوت آماری معناداری ایجاد شده بود (0.05>P). اما بین نمرۀ نگرش آیده‌ال گرایانه دو گروه در پس‌آزمون تفاوت آماری معنی داری مشاهده نشد. نتیجه گیری: باتوجه به نتایج بدست آمده، آموزش مفاهیم نه گانه شخصیتی انیگرام برنگرش به ازدواج و ملاک‌های همسرگزینی دختران مؤثر می‌باشد.

Title: تاثیر برنامه آموزشی مبتنی بر تئوری انگیزش محافظت بر انجام تست پاپ اسمیر در زنان مراجعه کننده به مراکز بهداشتی درمانی شهر فسا

http://jech.umsha.ac.ir/article-1-236-fa.html

سابقه و هدف: با وجود اهمیت پاپ اسمیر در کاهش میزان بروز سرطان دهانه رحم و مرگ ناشی از آن، این سرطان ﻫﻨﻮز  هم یکی از علل اصلی مرگ و میر در میان زنان در سراسر جهان است. این مطالعه با هدف تعیین اثربخشی برنامه آموزشی متنی بر تئوری انگیزش محافظت ﺑﺮ اﻧﺠﺎم آزﻣﺎﻳﺶ ﭘﺎپ  اﺳﻤﻴﺮ در ﺑﻴﻦ زﻧﺎن اجرا گردید. مواد و روش‌ها: این مطالعه نیمه‌تجربی در میان 200 زن مراجعه کننده به مراکز بهداشتی درمانی شهر فسا (100 نفر گروه آزمون و 100 نفر گروه کنترل) انجام شد. اﺑﺰار ﮔﺮدآوری اﻃﻼﻋﺎت ﭘﺮﺳﺸﻨﺎﻣﻪای مشتمل بر ﻣﺘﻐﻴﺮﻫﺎی جمعیت شناختی و سازه‌های تئوری انگیزش محافظت بود که توسط هر دو گروه قبل و 2 ماه بعد از مداخله تکمیل گردید. برنامه آموزشی طی 4 جلسه با شیوه بحث گروهی و ارائه پمفلت آموزشی برای گروه مداخله اجرا شد. داده‌ها توسط نسخه 22 نرم افزار آماری SPSS و با آزمون‌های آماری ﻛﺎی دو، ﺗﻲ‌ﻣﺴﺘﻘﻞ و تی‌زوجی تجزیه و تحلیل شد. یافته‌ها: نتایج این مطالعه نشان داد که میانگین نمرات تمام سازه‌های تئوری انگیزش محافظت بجز سازه کارآیی پاسخ (0.228=P) در گروه آزمون نسبت به گروه کنترل بعد از مداخله آموزشی افزایش یافت (0.001>P). همچنین پس از مداخله آموزشی، میزان انجام پاپ اسمیر در زنان گروه مداخله 58 درصد افزایش یافت. نتیجه‌گیری: نتایج مطالعه اثربخشی مداخله آموزشی بر اساس تئوری انگیزش محافظت در جهت ارتقاء رفتار پاپ اسمیر را نشان می‌دهد. لذا طراحی مداخلات آموزشی براساس این تئوری در جهت آموزش و ارتقای سلامت جامعه و به ویژه زنان پیشنهاد می‌گردد.

Title: بررسی ارتباط بین سواد سلامت با آگاهی و نگرش نسبت به مضرات مصرف سیگار در دانشجویان

http://jech.umsha.ac.ir/article-1-243-fa.html

سابقه و هدف: بین سواد سلامت و وضعیت مصرف سیگار ارتباط وجود دارد. با توجه به روند صعودی شیوع مصرف سیگار و همچنین نقش الگویی دانشجویان علوم پزشکی در کشور، این پژوهش با هدف تعیین ارتباط بین سواد سلامت با آگاهی و نگرش در زمینه مضرات مصرف سیگار انجام گردید. مواد و روش‌‌ها: این مطالعه مقطعی و از نوع همبستگی بود. مطالعه حاضر در سال 1395 و در میان 340 نفر از دانشجویان ساکن در خوابگاه‌های دانشگاه علوم پزشکی شهید بهشتی تهران که به روش نمونه‌گیری خوشه‌ای یک مرحله‌ای انتخاب شده بودند، انجام شد. ابزار گردآوری اطلاعات شامل پرسشنامه سواد سلامت جمعیت شهری 18 تا 65 ساله ایرانی (Health Literacy for Iranian Adults: HELIA) و پرسشنامه محقق‎ساخته آگاهی و نگرش در خصوص مضرات مصرف سیگار بود. سپس داده‌ها در نرم‌افزار SPSS16  و با استفاده از آماره‌های توصیفی و آزمون‌های Chi-Square و ضریب همبستگی پیرسون مورد تجزیه و تحلیل قرار گرفتند. یافته‌ها: میانگین و انحراف معیار نمره‌های سواد سلامت، آگاهی و نگرش به‌ترتیب 14.12±70.52، 1.62±16.65 و 10.33±53.03 از 100 بود. بین آگاهی و نگرش در خصوص مضرات مصرف سیگار و همچنین بین سواد سلامت و آگاهی و نگرش در خصوص مضرات مصرف سیگار ارتباط مستقیم و معنی‌داری مشاهده شد (0.001>P). نتیجه‌گیری: با توجه به ارتباط سواد سلامت با آگاهی و نگرش در خصوص مضرات مصرف سیگار، بایستی در مطالعات آتی به بررسی تأثیر سطوح مختلف سواد سلامت بر مصرف سیگار پرداخته شود.

Title: بررسی تأثیر آموزش هوش هیجانی بر مبنای تئوری حمایت اجتماعی در کاهش استرس درک‌شده و ارتقاء مهارت‌های ارتباطی دانش‌آموزان پسر مقطع دبیرستان شهر همدان

http://jech.umsha.ac.ir/article-1-234-fa.html

سابقه و هدف: تحقیقات نشان داده است که 80 درصد موفقیت افراد مربوط به مهارت‏هایی است که به نوعی با هوش هیجانی در ارتباط می‏باشد؛ بنابراین مطالعه حاضر با هدف بررسی تأثیر آموزش هوش هیجانی بر مبنای تئوری حمایت اجتماعی در کاهش استرس درک‌شده و ارتقاء مهارت‏های ارتباطی دانش‏آموزان پسر دبیرستانی شهر همدان انجام شد. مواد و روش‌‌ها: در این مطالعه مداخله‌ای، 100 دانش‏آموز پسر دبیرستانی به‌صورت تصادفی به دو گروه آزمون و کنترل (هر گروه 50 نفر) تقسیم شده بودند. ابزار گردآوری اطلاعات شامل پرسشنامه حمایت اجتماعی (Social Support Scales: SSS)، هوش هیجانی Schutte Self-Report Emotional Intelligence Test، مهارت‏های ارتباطی  Communication Skills Inventory و پرسشنامه استرس درک‌شده  Perceived Stress Scale بود. برنامه مداخله‌ای شامل پنج جلسه‏ آموزشی جهت دانش‏آموزان و دو جلسه جهت والدین و مربیان بود که در مدت‌زمان یک ماه برای شرکت‌کنندگان گروه آزمون به مرحله اجرا درآمد و دو گروه آزمون و کنترل یک ماه و دو ماه پس از مداخله آموزشی مورد پیگیری قرار گرفتند. داده‏ها با نرم‌افزار 16 SPSS، آزمون‌های Chi-Square، T زوجی، T مستقل و آنالیز طرح اندازه‏های تکراری تجزیه و تحلیل گردید. یافته‌ها: بعد از اجرای برنامه مداخله‌ای، اختلاف معنی‏داری بین میانگین نمرات دانش‏آموزان در سازه‏های تئوری حمایت اجتماعی، هوش هیجانی، مهارت‏های ارتباطی و استرس درک‌شده بین گروه‌های آزمون و کنترل مشاهده گردید (0.05>P). نتیجه‌گیری: یافته‏های این پژوهش نشان داد آموزش هوش هیجانی بر مبنای حمایت اجتماعی در کاهش استرس و بهبود مهارت‌های ارتباطی دانش‌آموزان مؤثر بوده است؛ بنابراین استفاده از این چارچوب نظری برای طراحی و ارزیابی مداخلات آموزشی پیشنهاد می‌گردد.

Title: پیش‌بینی اعتیاد به اینترنت و اضطراب اجتماعی براساس سبک‌های فرزندپروری در نوجوانان شهر سبزوار

http://jech.umsha.ac.ir/article-1-257-fa.html

سابقه و هدف: سبک‌های فرزندپروری یکی از مهم‌ترین عوامل مؤثر در شکل‌گیری رفتار و شخصیت نوجوانان است. از این رو هدف از پژوهش حاضر، پیش‌بینی اعتیاد به اینترنت و اضطراب اجتماعی براساس سبک‌های فرزندپروری بود. مواد و روش‌‌ها: این پژوهش، یک مطالعه توصیفی و از نوع همبستگی بود که در سال 1395 انجام شد. بدین منظور، تعداد 400 نفر از دانش‌آموزان مدارس متوسطه شهرستان سبزوار به ‌روش نمونه‌گیری خوشه‌ای چندمرحله‌ای انتخاب شدند. ابزار گردآوری اطلاعات شامل: پرسشنامه‌ اعتیاد به اینترنت Jung (IAT: Implicit-Association Test)، مقیاس اضطراب اجتماعی (Social Anxiety Scale for Adolescents SAS-A:) و پرسشنامه سبک‌های فرزندپروری Baumrind (Questionnaire Parenting Style BPSQ: Baumrind) بود. داده‌ها با استفاده از ضریب همبستگی پیرسون و رگرسیون چندگانه در نرم‌افزار 22 SPSS تحلیل گردید. یافته‌ها: نتایج نشان داد که همبستگی مثبت و معنی‌داری بین سبک‌های فرزندپروری استبدادی و آزادگذار با اعتیاد به اینترنت (102/0r=، 210/0r=) و اضطراب اجتماعی (576/0=، 517/0r=r) وجود دارد. همچنین همبستگی منفی و معنی‌داری بین سبک مقتدرانه با اعتیاد به اینترنت (0.291-=r) و اضطراب اجتماعی (0.263-=r) مشاهده شد. علاوه بر آن، نتایج رگرسیون چندگانه نشان داد که سبک‌های فرزندپروری پیش‌بینی‌کننده‌ی معنی‌دار اعتیاد به اینترنت و اضطراب اجتماعی بوده و به‌ترتیب 39 و 35 درصد از واریانس آن‌ها را تبیین می‌نمود. نتیجه‌گیری: با توجه به نتایج به‌دست آمده، می‌توان با استفاده از روش‌های تربیتی مناسب و ایجاد برنامه‌ریزی‌های کاربردی، اعتیاد به اینترنت را پیش‌بینی کرد و ارتباط مثبت با دیگران را در افراد ارتقاء داد.

Title: بررسی عوامل مرتبط با عملکرد آرایشگران مرد نسبت به پیشگیری از بیماری ایدز براساس مدل اعتقاد بهداشتی در شهر فسا

http://jech.umsha.ac.ir/article-1-76-fa.html

سابقه و هدف: تعداد مبتلایان به بیماری ایدز در کشور رو به افزایش است و یکی از راه‌های انتقال بیماری، آلودگی وسایل تیز و برنده‌ای است که بدون ضدعفونی برای افراد مختلف استفاده می‌شود. مطالعه حاضر با هدف بررسی عملکرد آرایشگران مرد نسبت به پیشگیری از بیماری ایدز براساس مدل اعتقاد بهداشتی در شهر فسا انجام گردید. مواد و روش‌‌ها: این مطالعه توصیفی- مقطعی در سال 1393 روی 220 نفر از آرایشگران مرد شهر فسا که به‌صورت تصادفی چند مرحله‌ای انتخاب شده بودند، انجام شد. ابزار گردآوری داده‌ها شامل: پرسشنامه‌ی محقق ساخته‌ای بود که مشتمل بر اطلاعات جمعیت‌شناختی، آگاهی و سازه‌های مدل اعتقاد بهداشتی بود و به روش خودگزارش‌دهی تکمیل گردید. داده‌ها توسط نرم‌افزار 18 SPSS از طریق آزمون رگرسیون خطی تجزیه و تحلیل شدند. یافته‌ها: براساس نتایج پژوهش حاضر آرایشگران آگاهی خوب، حساسیت درک‌شده متوسط، شدت درک‌شده کم، منافع درک‌شده زیاد، موانع درک‌شده کم و عملکرد خوبی داشتند. سازه‌های آگاهی، حساسیت درک‌شده و منافع درک‌شده پیشگویی‌کننده‌ی عملکرد آرایشگران در خصوص پیشگیری از ایدز بودند. به‌طور کلی متغیرهای مورد بررسی، 31.5 درصد واریانس عملکرد آرایشگران را پیشگویی می‌کردند. نتیجه‌گیری: با توجه به نتایج این مطالعه، می‌توان با برنامه‌ریزی مناسب در خصوص افزایش آگاهی، حساسیت درک‌شده و منافع درک‌شده نسبت به بیماری ایدز عملکرد آرایشگران را ارتقاء داد و این خود در پیشگیری و کنترل بیماری ایدز اهمیت به‌سزایی دارد.
